# Outstanding Seizure Characteristics With Etomidate and Ketofol

**DOI:** 10.1192/j.eurpsy.2022.1901

**Published:** 2022-09-01

**Authors:** H.C. Özden, S.C. Gurel

**Affiliations:** Hacettepe University Faculty Of Medicine, Department Of Psychiatry, Ankara, Turkey

**Keywords:** anaesthesia, etomidate, Electroconvulsive therapy, ketofol

## Abstract

**Introduction:**

Electroconvulsive therapy (ECT) is administered following general anaesthetic induction with methohexital, thiopental, etomidate, alfentanil, remifentanil, propofol or ketamine. One approach for idealizing the induction anaesthesia for ECT is combining two agents (e.g. ketamine-propofol) with synergistic anaesthetic properties and non-additive anticonvulsive and hyperdynamic effects.

**Objectives:**

To establish any superiority between ketamine-propofol (ketofol) combination and etomidate in terms of seizure characteristics and hemodynamic measures.

**Methods:**

We have combined our previous case series (etomidate vs thiopental) with new data regarding propofol and ketofol. ECT stimulus duration, stimulus frequency, the stimulus charge applied, duration of central seizure time, number of stimulation trials, plus anaesthetic used in the individual sessions were retrieved. A total number of 1092 sessions (239 sessions with etomidate, 233 with thiopental, 275 with propofol, and 345 with ketofol induction) were included in the linear mixed-effects model analysis.

**Results:**

Etomidate was superior in terms of seizure duration compared with thiopental. There was no significant difference in seizure durations between ketofol, propofol and thiopental, however, number of failed stimulation trials within a session increased significantly with propofol use compared with etomidate and ketofol. The required amount of charge (stimulation dosage) was significantly lower when ketofol was used, compared with thiopental. Additionally, within the ketofol sessions only the propofol dose significantly increased the amount of required dose.

**Conclusions:**

Etomidate and ketofol displayed certain superiorities in terms of seizure characteristics when used as induction anaesthetics for ECT. Therefore, both etomidate and ketamine used in combination with propofol may be considered to be the gold standards of ECT anaesthesia.

**Disclosure:**

No significant relationships.

